# Integrative Analyses of Mitophagy-Related Genes and Mechanisms Associated with Type 2 Diabetes in Muscle Tissue

**DOI:** 10.3390/cimb46090619

**Published:** 2024-09-18

**Authors:** Wangjia Mao, Guannan Zong, Yuan Gao, Shen Qu, Xiaoyun Cheng

**Affiliations:** 1 Department of Endocrinology and Metabolism, Division of Metabolic Surgery for Obesity and Diabetes, Shanghai Tenth People’s Hospital, Institute of Obesity, Institute of Thyroid Diseases, Shanghai Center of Thyroid Diseases, School of Medicine, Tongji University, Shanghai 200072, China; 2131118@tongji.edu.cn; 2 Department of Endocrinology and Metabolism, Tongji Hospital, School of Medicine, Tongji University, Shanghai 200092, China; zgn897580@163.com; 3 School of Medicine, Tongji University, Shanghai 200092, China; 13181961861@163.com

**Keywords:** Type 2 diabetes, autophagy-related genes, mitophagy, molecular mechanisms, pathogenesis

## Abstract

Type 2 diabetes (T2D) represents the most prevalent metabolic condition that is primarily distinguished by a range of metabolic imbalances, including hyperglycemia, hyperlipidemia, and insulin resistance (IR). Currently, mitophagy has become increasingly recognized as an important process involved in the pathogenesis and progression of T2D. Therefore, it is very important to explore the role of mitochondrial damage and autophagy-related genes in T2D. This study investigated the role of mitophagy in the development of T2D, and 12 MRHGs associated with T2D were identified using bioinformatic analysis and machine learning methods. Our findings provide the first insight into mitophagy-related genes and their mechanisms in T2D. This study aimed to investigate possible molecular targets for therapy and the underlying mechanisms involved in T2D. This information might be useful to further elucidate the pathogenesis of T2D-related diseases and identify more optimal therapeutic approaches.

## 1. Introduction

Recently, with rising living standards and changes in lifestyle, the frequency of diabetes is increasing day by day, and it has become a worldwide disease that seriously endangers people’s health. The 2021 IDF Global Diabetes Map indicated that the global count of adult diabetics reached 537 million in 2021, and this number is still increasing. China is the largest country with diabetes, with 141 million adult diabetic patients [1]. T2D is linked to the presence of abdominal obesity, a precursor state known as prediabetes, as well as abnormal blood lipid levels referred to as dyslipidemia [2,3]. It is distinguished by elevated blood glucose levels, impaired function of beta cells, and a reduced sensitivity to insulin [4]. Islet β cell dysfunction plays a key role in the pathogenesis of T2D. Among the many factors leading to islet β-cell dysfunction, islet β-cell apoptosis plays a key role.

Mitochondria, vital double-membraned organelles within cells, play a pivotal role in numerous essential processes, such as energy production, metabolic regulation, maintaining redox balance, cell specialization, and ion balance. As such, they serve as the primary controllers of cellular life and death. These organelles continuously produce reactive oxygen species (ROS), highly reactive byproducts of oxygen metabolism, which function as signaling molecules crucial for regulating cell survival, mitochondrial functionality, and insulin sensitivity [5]. Glucose homeostasis depends on mitochondria (MT), suggesting that MT are involved in DM [6]. There is evidence that mitochondrial bioenergy deficiency can lead to impaired glucose metabolism [7]. MT act as engines to provide energy for cell metabolism. Mitophagy is a process that eliminates and recycles defective mitochondria while also managing the creation of new, fully operational mitochondria. This mechanism helps to maintain the overall health and functionality of mitochondrial activities [8]. The onset of oxidative stress and mitochondrial impairment represent early stages in the progression of chronic diseases. These initial events can subsequently trigger alterations in β-cell function, synaptic malfunction, and the disruption of energy homeostasis [9,10,11,12,13,14]. Given the intricate interplay among mitochondria, oxidative stress, and metabolism, as well as the pivotal role of autophagy/mitophagy in managing T2D, it is understandable that there has been significant interest in manipulating these processes as a promising therapeutic strategy.

To better understand T2D, it is necessary to study the pathogenesis of T2D in order to develop effective treatments. In particular, mitochondrial dynamics plays a key role in the pathophysiology of diabetes [15]. This article thoroughly explores how mitochondrial damage contributes to the development and advancement of T2D, with a particular emphasis on mitophagy. It also examines the mechanisms that could be pivotal in using mitophagy as a potential therapeutic approach for managing and preventing T2D.

## 2. Materials and Methods

### 2.1. Data Collection and Preprocessing

The datasets GSE166467 [16], GSE166652 [16], and GSE166502 [16] related toT2D were obtained from the Gene Expression Omnibus (GEO) (http://www.ncbi.nlm.nih.gov/geo, accessed on 1 August 2023) database using R version 4.2.0 [17]. The details and specific information are shown in Table 1. The datasets GSE166467 and GSE166652 were used as test sets. The chip platforms were GPL10558 and GPL13534. The dataset GSE166502 was used as the validation set. The sample was from *Homo sapiens* muscle tissue.

A compilation of mitophagy-related genes (MRGs) was gathered from both the GeneCards [18] database and the Molecular Signatures Database (MSigDB) [19]. A total of 2377 MRGs were obtained after merging and deduplication, and the specific information is shown in Table S1.

The R package sva [20] was employed to remove batch effects from the datasets GSE166467 and GSE166652, resulting in a combined GEO dataset. Subsequently, both the consolidated GEO dataset and the GSE166502 dataset underwent standardization procedures using the R package limma [21]. Additionally, the annotation probes are subjected to a process of standardization and normalization to ensure uniformity and comparability across the datasets.

### 2.2. Identification of MRDEGs

According to GEO dataset, the samples were divided into the T2D and control groups. The differences in gene expression between the T2D and control groups were analyzed using the R package limma [18]. The criteria for differentially expressed genes (DEGs) are set as |logFC| > 0.00 and adj.*p* < 0.05. In order to obtain mitophagy-related differentially expressed genes (MRDEGs) involved in T2D, the intersection of all DEGs (|logFC| > 0.00 and adj.*p* < 0.05) and MRGs identified through differential analysis of the integrated GEO dataset was obtained from a Venn diagram. The findings from differential analysis were presented using the R package ggplot2 to create a volcano plot, the R package pheatmap to draw a heat map, and the R package RCircos [22] to draw a chromosome localization map.

### 2.3. Functional Annotation Coupled with Pathway Enrichment Assessment 

Gene Ontology (GO) [23] analysis is a prevalent approach for conducting large-scale functional enrichment investigations that encompass both biological processes (BP) and molecular functions (MF). The Kyoto Encyclopedia of Genes and Genomes (KEGG) [24] serves as a ubiquitous resource for cataloging and accessing knowledge pertaining to genomic sequences and biological pathway networks, diseases, and drugs. The GO and KEGG annotation analysis of MRDEGs was conducted utilizing the R package known as clusterProfiler [25]. The item screening criteria were adj.*p* < 0.05 and FDR value (q value) < 0.25, which was considered statistically significant. The adj.*p* correction method was the Benjamini-Hochberg (BH) method.

### 2.4. Gene Set Enrichment Analysis (GSEA)

Gene set enrichment analysis (GSEA) [26] assesses the tendency of genes within a predefined gene set to cluster in a gene table that has been ordered based on their phenotypic correlation, thereby enabling the evaluation of their collective contribution to a particular phenotype. In this study, the integrated GEO dataset was divided into two groups: T2D and control. Then, the R package clusterProfiler was used to perform GSEA on all genes in the integrated GEO dataset according to the logFC value. The GSEA parameters are set as follows: a seed value of 2020 for reproducibility, a total of 1000 permutations for statistical robustness, a minimum requirement of 10 genes per gene set to ensure sufficient data for analysis, and a maximum limit of 500 genes per set to manage computational complexity. The MSigDB [19] database provided the c2.cp.all.v2022.1.Hs.symbols.gmt [All Canonical Pathways] gene set, encompassing all 3050 canonical pathways, which was subsequently employed for conducting GSEA. The filtering standards for GSEA were adj.*p* < 0.05 and FDR value (q value) < 0.25.

Similarly, utilizing the median value of the LASSO risk score as a cutoff, the T2D samples were categorized into two distinct groups: a high-risk group and a low-risk group. Then, the R package clusterProfiler was used to perform GSEA on all genes in the T2D samples according to the logFC value. For the GSEA analysis, the following parameters were employed: a seed value of 2020 for reproducibility, 1000 permutations for statistical robustness, a minimum gene count of 10 per gene set to ensure adequate representation, and a maximum gene count of 500 per set to manage computational complexity. The c2.all.v2022.1.Hs.symbols.gmt [Curated/Pathway] (6449) gene set was obtained from the MSigDB [16] database for GSEA. The screening criteria for GSEA were adj.*p* < 0.05 and FDR value (q value) < 0.25.

### 2.5. Gene Set Variation Analysis (GSVA)

Gene set variation analysis (GSVA) [27] is an unsupervised, non-parametric method used to assess gene set enrichment by converting the gene expression matrix across different samples into a gene set expression matrix. To determine if various pathways are enriched across different samples. The c2.all.v7.5.1.symbols.gmt gene set was obtained from the MSigDB database, and the GSVA was performed on all genes in the integrated GEO dataset to calculate the functional enrichment differences between the two groups of T2D and control. The screening criteria of GSVA were |logFC| > 0.00 and adj.*p* < 0.05.

Similarly, T2D samples were categorized into the high-risk and low-risk groups according to the median value of LASSO risk score. The c2.all.v7.5.1.symbols.gmt gene set was retrieved from the MSigDB database and GSVA was applied to all genes in T2D samples. The functional enrichment differences between the high-risk and low-risk groups were then assessed. The screening criteria of GSVA were |logFC| > 0.30 and *p* value < 0.05.

### 2.6. Development of Diagnostic Model for T2D

In order to obtain the diagnostic model of T2D using the integrated GEO dataset, the MRDEGs were analyzed using logistic regression analysis. When the dependent variable is a binary variable, that is, the T2D and control groups, logistic regression was employed to examine the relationship between predictor and outcome variables. The MRDEGs were screened using adj.*p* < 0.05 as the standard, and a logistic regression model was developed. A forest plot was utilized to visually represent the molecular expression of MRDEGs that were incorporated into the logistic regression model.

Then, based on the MRDEGs contained in the logistic regression model, the SVM model was constructed using the support vector machine (SVM) [28] algorithm, and the MRDEGs were selected based on the gene count that achieved the highest accuracy and the lowest error rate.

Finally, LASSO regression analysis was performed using the R package glmnet [29] with set.seed (500) as a parameter based on the MRDEGs contained in the SVM model. LASSO regression analysis is derived from linear regression techniques. By increasing the penalty term (the absolute value of lambda × slope), the over-fitting of the model is reduced, and the generalization ability of the model is improved. The outcomes of LASSO regression analysis are visualized using a diagnostic model diagram and variable trajectory diagram. The results of LASSO regression analysis were used to generate the diagnostic model of T2D, which contained MRDEGs as mitophagy-related hub genes (MRHGs).

### 2.7. Validation of the Diagnostic Model for T2D

The nomogram [30] is a graphical tool where a series of separate line segments illustrate the functional relationship among several independent variables on a rectangular coordinate system. Drawing from the findings of the LASSO regression analysis, the nomogram was drawn using the R package rms to show the relationship between MRHGs. The calibration curve was generated using calibration analysis to assess the accuracy and resolution of the diagnostic model of T2D based on the results of LASSO regression analysis, and the decision curve analysis (DCA) [31] was performed using R package ggDCA based on the MRHGs in the integrated GEO dataset. DCA is a straightforward approach for assessing clinical predictive models, diagnostic tests, and molecular markers. Finally, the R package pROC was used to draw the ROC curve with the integrated GEO dataset and calculate the area under the ROC curve (area under the curve, AUC) to evaluate the diagnostic effect of the LASSO risk score on the occurrence of T2D. The AUC of ROC curves typically ranges from 0.5 to 1. An AUC closer to 1 indicates a better diagnostic performance. When the AUC falls between 0.5 and 0.7, it reflects low accuracy. An AUC between 0.7 and 0.9 indicates moderate accuracy. An AUC above 0.9 signifies high accuracy. The LASSO risks is calculated as follows:

$$riskScore=\sum_{i}Coefficient\;\left({gene}_{i}\right)\times mRNA\;Expression\;({gene}_{i})$$

Similarly, the accuracy and resolution of the diagnostic model of T2D were verified using the dataset GSE166502. First, the nomogram was drawn based on the results of LASSO regression analysis using the R package rms to show the interrelationship of MRHGs in the dataset GSE166502. Secondly, the LASSO risk score of dataset GSE166502 was calculated based on the expression of MRHGs in the dataset and the LASSO coefficient of T2D diagnostic model. Then, a calibration curve was drawn based on calibration analysis, and the DCA results are presented using the R package ggDCA based on the MRHGs in the dataset GSE166502. Finally, the R package pROC was used to draw the ROC curve for the dataset GSE166502 and calculate the area under the ROC curve (AUC) to evaluate the diagnostic effect of the LASSO risk score on the occurrence of T2D.

### 2.8. Expression Difference and Correlation Analysis

First, in order to further investigate the variations in expression of MRHGs between the T2D and control groups within the combined GEO dataset and the dataset GSE166502, a group comparison diagram was drawn based on the expression of MRHGs.

Then, in order to further explore the correlation between MRHGs, the Spearman algorithm was employed to examine the relationship between the expression of MRHGs in the integrated GEO dataset and the dataset GSE166502. The results of correlation analysis were displayed using the R package RCircos [22].

### 2.9. Relationship between Immune Cell Infiltration and Mitophagy-Related Genes

CIBERSORT [32] uses linear support vector regression principles to deconvolution the transcriptome expression matrix, allowing for the estimation of immune cell composition and abundance in mixed cells. Using the CIBERSORT algorithm, the LM22 feature gene matrix was integrated, and the data were filtered to include only the immune cell enrichment fraction greater than zero. The final results of the immune cell infiltration matrix are obtained. Finally, the correlation heat map was drawn using the R package pheatmap to show the correlation analysis results of the LM22 immune cells themselves and MRHGs with LM22 immune cells.

### 2.10. Construction of the Regulatory Network and Prediction of Protein Domains

Transcription factors (TFs) control gene expression by interacting with MRHGs during the post-transcriptional phase. The regulatory effect of transcription factors on MRHGs was analyzed based on the transcription factors retrieved from the ChIPBase database [33], and the mRNA–TF regulatory network was visualized using Cytoscape software (Version 3.9.1).

In addition, miRNA significantly influences biological development and evolution through its regulatory functions. It could regulate various target genes, and a single target gene might be influenced by multiple miRNAs. To explore the relationship between MRHGs and miRNAs, miRNAs associated with MRHGs were sourced from the starBase v2.0 database [34], and the mRNA–miRNA regulatory network was visualized using Cytoscape software.

The protein–protein interaction (PPI) network consists of proteins interconnected through various interactions. The STRING database is a database that searches for known proteins and predicts PPIs. In this study, the STRING database [35] was used to construct a PPI network related to mitophagy-related hub genes based on the mitophagy-related hub genes with a minimum interaction score set above 0.400 (medium confidence threshold: (0.400)). Cytoscape software [36] was used to visually predict the associated MRHGs.

### 2.11. Statistical Analysis

All the data processing and analysis in this study were conducted using R software (Version 4.2.0), and the continuous variables are displayed in as mean ± standard deviation. The Wilcox test was used for comparisons between the two groups. If it is not specifically specified, Spearman correlation analysis was used to compute the correlation coefficients between different molecules, with significance determined by an adjusted *p*-value of <0.05.

## 3. Results

### 3.1. Analysis of Differentially Expressed Genes Related to Mitophagy

**Figure 1:**
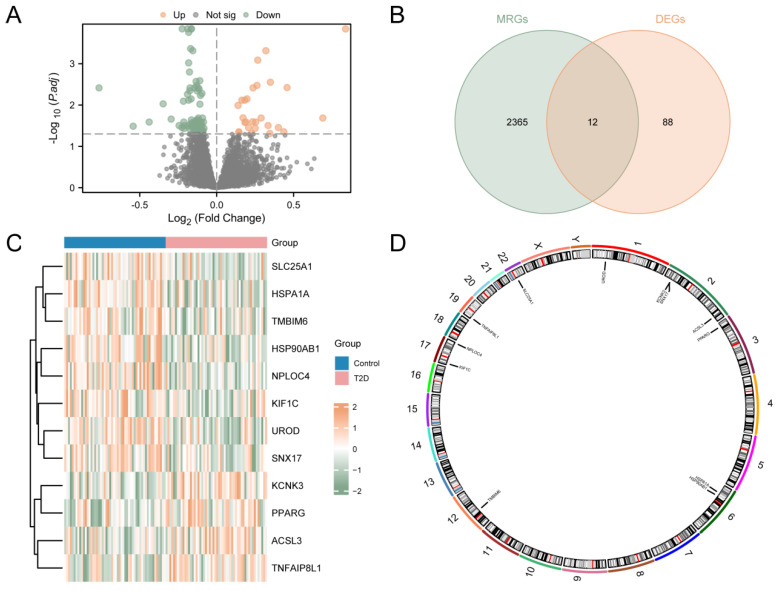
Differential Gene Expression Analysis for Combined Datasets. (**A**) Volcano map analysis of differentially expressed genes between the T2D group and the control group using the integrated GEO dataset. (**B**) Venn diagram of DEGs and MRGs from the integrated GEO dataset. (**C**) MRDEGs in the integrated GEO dataset. (**D**) Chromosomal localization of MRDEGs. T2D, Type 2 Diabetes; DEGs, Differentially Expressed Genes; MRGs, Mitophagy-Related Genes; MRDEGs, Mitophagy-Related Differentially Expressed Genes. Blue represents the control group, and pink represents the T2D group.

The study was designed as indicated in the flow chart (Supplementary Figure S1). The process used to merge the T2D datasets is shown in Supplementary Figure S2. In order to analyze the difference in the gene expression value between the T2D and control groups in the integrated GEO dataset, the data were analyzed using the R package limma to obtain the differentially expressed genes of the two groups of data. The results are as follows. From the GEO dataset, a total of 100 genes met the criteria (|logFC| > 0.00 and adj.*p* < 0.05), including 27 up-regulated genes (logFC > 0.00 and adj.*p* < 0.05) and 73 down-regulated genes (logFC < 0.00 and adj.*p* < 0.05). A volcano map was generated based on the analysis (Figure 1A). A total of 12 MRDEGs were obtained and shown in the Venn diagram (Figure 1B), including KCNK3, ACSL3, PPARG, TNFAIP8L1, HSP90AB1, HSPA1A, UROD, NPLOC4, TMBIM6, SLC25A1, KIF1C, and SNX17. Then, the R package pheatmap was used to display the analysis results (Figure 1C). Finally, the positions of the 12 MRDEGs on human chromosomes were analyzed using the R package RCircos. The chromosome localization map is shown in Figure 1D. Chromosome mapping showed that most MRDEGs were located on chromosome 2, including KCNK3, SNX17, and ACSL3.

### 3.2. GO and KEGG Pathway Enrichment Analysis 

**Figure 2:**
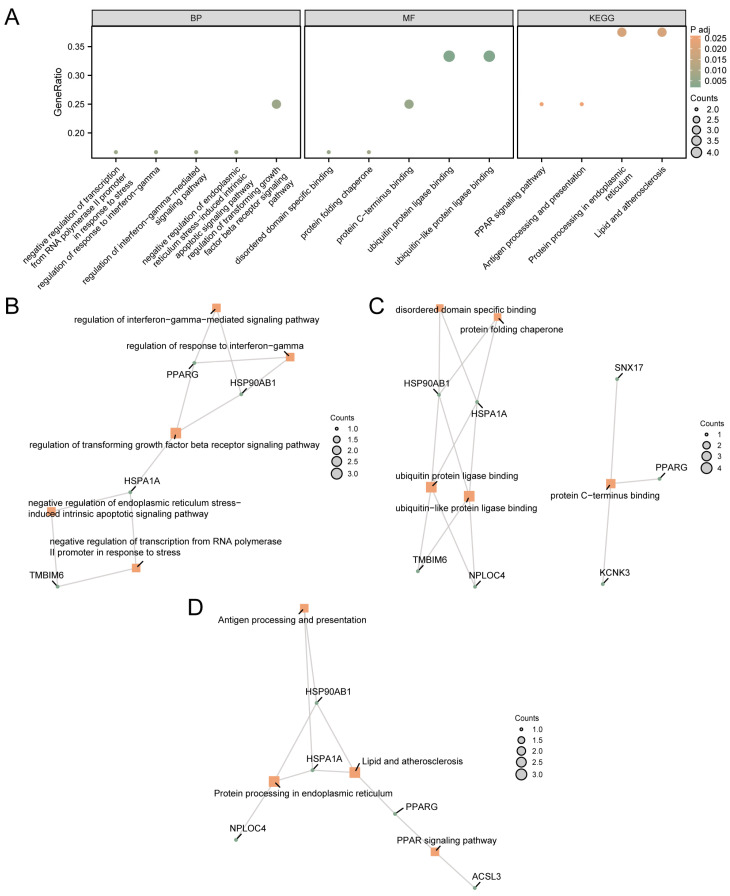
GO and KEGG Enrichment Analysis for MRDEGs. (**A**) GO and KEGG pathway enrichment analysis results of MRDEGs in a bubble map display: BP, MF, and biological pathway. The abscissa is GO terms and KEGG terms. The results of GO and KEGG pathway enrichment analysis of MRDEGs based on BP (**B**), MF (**C**), and KEGG (**D**). The orange node represents the item, the green node represents the molecule, and the connection represents the relationship between the item and the molecule. MRDEGs, Mitophagy-Related Differentially Expressed Genes; GO, Gene Ontology; KEGG, Kyoto Encyclopedia of Genes and Genomes; BP, Biological Process; MF, Molecular Function. The screening criteria for GO and KEGG pathway enrichment analysis were adj.*p* < 0.05 and FDR value (q value) < 0.25.

These 12 MRDEGs were used for GO and KEGG pathway enrichment analysis. The specific results are shown in Supplementary Table S2. The results showed that 12 MRDEGs were mainly enriched in negative regulation of transcription from the RNA polymerase II promoter in response to stress, regulation of the response to interferon-gamma, and regulation of interferon-gamma-mediated signaling pathways. Biological processes (BP) such as regulation of the transforming growth factor beta receptor signaling pathway, negative regulation of endoplasmic reticulum stress-induced intrinsic apoptotic signaling pathway, ubiquitin protein ligase binding, ubiquitin-like protein ligase binding, protein C-terminus binding, disordered domain-specific binding, and protein folding chaperone were enriched. In addition, biological pathways such as protein processing in the endoplasmic reticulum, lipids and atherosclerosis, the PPAR signaling pathway, and antigen processing and presentation were enriched. The results of GO and KEGG pathway enrichment analysis were visualized using bubble map (Figure 2A).

The network diagram of MF and BP was shown (Figure 2B–D). The connection indicates the corresponding molecule and the corresponding entry annotation. The larger the node is, the more molecules the entry contains.

### 3.3. Gene Set Enrichment Analysis (GSEA) of Integrated GEO Datasets

**Figure 3:**
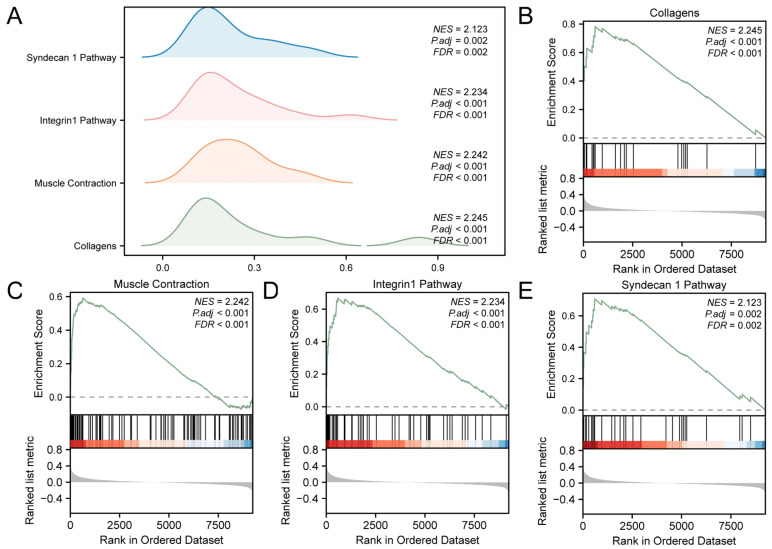
GSEA for Combined Datasets. (**A**) GSEA results for the integrated GEO datasets presented as a mountain map display of the four biological functions. (**B**–**E**). GSEA showed that T2D significantly affected collagens (**B**), muscle contraction (**C**), integrin 1 pathway (**D**) and syndecan 1 pathway (**E**). T2D, Type 2 Diabetes; GSEA, Gene Set Enrichment Analysis. The screening criteria of GSEA were adj.*p* < 0.05 and FDR value (q value) < 0.25.

The links among the biological processes involved, the cellular components impacted, and the molecular functions performed (Figure 3A) are shown in Supplementary Table S3. The results showed that all genes in the GEO were significantly enriched in biological-related functions and signaling pathways, such as collagens (Figure 3B), muscle contraction (Figure 3C), integrin 1 pathway (Figure 3D), and syndecan 1 pathway (Figure 3E).

### 3.4. Gene Set Variation Analysis (GSVA) of the Integrated GEO Dataset

GSVA was conducted on the entire set of genes in the integrated GEO dataset. The specific information is shown in Supplementary Table S4. Pathways with |logFC| > 0.00 and adj.*p* < 0.05 were screened, and the results were presented in a group comparison box plot (Supplementary Figure S3A). The results of GSVA showed that 10 pathways were statistically significant in the T2D versus control group comparison (*p* value < 0.05). These pathways include genes targeted by miRNAs in adipocytes, rho GTPases activating rho-tekins and rho-philins, zamora Nos2 targets, Chiba response to TSA, SARS-CoV infections, P2Y receptors, Nikolsky breast cancer 20q11 amplicon, uptake and actions of bacterial toxins, autophagy, and purinergic signaling. According to GSVA, the differential expression of 10 pathways with |logFC| > 0.00 and adj.*p* < 0.05 between T2D and control groups was analyzed and visualized using heat maps (Supplementary Figure S3B).

### 3.5. Construction of a Diagnostic Model for T2D

**Figure 4:**
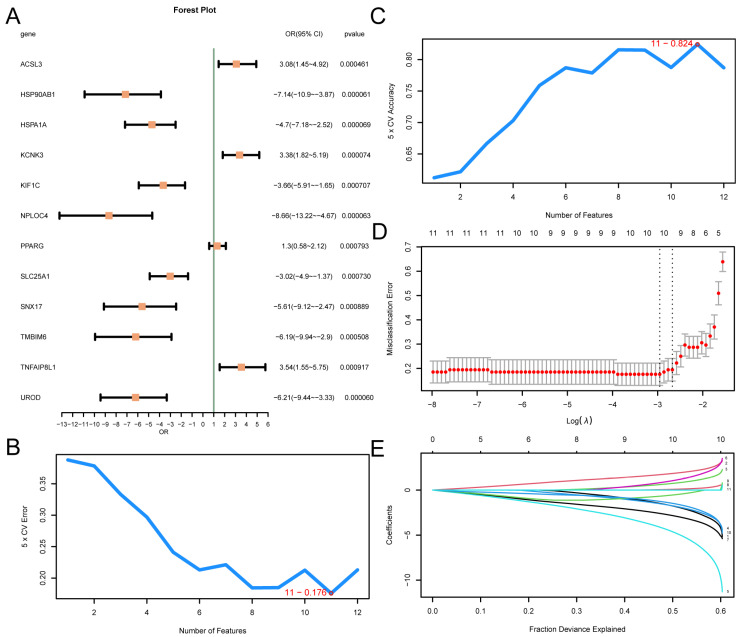
Diagnostic Model of T2D. (**A**) Forest plot of 12 MRDEGs included in the logistic regression model used to generate the diagnostic model of T2D. The number of genes with the lowest error rate (**B**) and the number of genes with the highest accuracy (**C**) obtained using the SVM algorithm are visually displayed. The diagnostic model diagram (**D**) and variable trajectory diagram (**E**) of the LASSO regression model. T2D, Type 2 Diabetes; SVM, Support Vector Machine; IncNodePurity, Increase in Node Purity; LASSO, Least Absolute Shrinkage and Selection Operator.

A logistic regression model was constructed based on 12 MRDEGs and visualized using a forest plot (Figure 4A). The results showed that the 12 MRDEGs included in the logistic regression model were statistically significant (*p* value < 0.05): KCNK3, ACSL3, PPARG, TNFAIP8L1, HSP90AB1, HSPA1A, UROD, NPLOC4, TMBIM6, SLC25A1, KIF1C, and SNX17. Then, the SVM model was constructed, and the number of genes with the minimal error rate (Figure 4B) and the highest level of accuracy (Figure 4C) was obtained. The findings revealed that the SVM model had the highest accuracy when the gene count was 11. The 11 MRDEGs included SLC25A1, KCNK3, PPARG, KIF1C, NPLOC4, TNFAIP8L1, UROD, ACSL3, HSPA1A, HSP90AB1, and TMBIM6. Then, based on the SVM model comprising 11 genes, the LASSO regression model was constructed using LASSO regression analysis, and the diagnosis model of T2D was generated. The LASSO regression model diagram (Figure 4D) and the LASSO variable trajectory diagram (Figure 4E) are visually displayed. The results showed that the LASSO regression model contained 10 MRDEGs, including SLC25A1, KCNK3, PPARG, KIF1C, NPLOC4, TNFAIP8L1, UROD, ACSL3, HSPA1A, and HSP90AB1.

### 3.6. Validation of the Diagnostic Model for T2D

A nomogram based on MRHGs was drawn to show the interrelationship of MRHGs in the integrated GEO datasets (Supplementary Figure S4A). The results showed that the MRHGs SLC25A1 and PPARG were significantly more effective than other variables in the diagnosis model of T2D. ACSL3 expression in the diagnostic model of T2D was significantly lower than that of other variables. The relationship between MRHGs in the dataset GSE166502 is shown in Supplementary Figure S4B. The results show that the expression of NPLOC4 was significantly more effective than other variables. The utility of HSPA1A expression was significantly lower than that of other variables.

Then, the calibration curve was created using calibration analysis to assess the model’s prediction accuracy by comparing the actual probability with the predicted probabilities in various scenarios (Supplementary Figure S4C). The calibration curve for the T2D diagnostic model indicates that the calibration line, which is represented by the dotted line, shows a slight deviation from the ideal model’s diagonal, but remains relatively close to it. Based on the MRHGs in the integrated GEO dataset, DCA was employed to assess the clinical utility of the T2D diagnostic model. The findings are illustrated in Supplementary Figure S4D. The results indicate that the model’s line remains consistently above both the all positive and all negative lines within a specific range, demonstrating higher net benefits and suggesting better performance of the model. In addition, the ROC curve was drawn using the R package pROC based on the LASSO risk score for the integrated GEO dataset. The ROC curve showed that the LASSO risk score for the integrated GEO dataset showed high accuracy between different groups (AUC > 0.9). The LASSO risk score is calculated as follows:

risk*Score* = SLC25A1 × (−1.4173) + KCNK3 × (1.6798) + PPARG × (0.5792) + KIF1C × (−1.4628) + NPLOC4

× (−4.0115) + TNFAIP8L1 × (0.7713) + UROD × (−2.5398) + ACSL3 × (0.0389) + HSPA1A × (−0.7096) +

HSP90AB1 × (−1.2119)

Similarly, the calibration curve was generated using calibration analysis to evaluate the model’s predictive accuracy by comparing the model’s predicted probabilities with the actual probabilities across various scenarios (Supplementary Figure S4F). In the calibration curve of the diagnostic model of T2D, the diagonal deviation between the calibration line is shown by the dotted line, and the ideal model is greater than the calibration curve of the integrated GEO dataset. Based on the MRHGs in GSE166502, the clinical utility of the T2D diagnostic model was assessed using DCA. The results are presented in Supplementary Figure S4G. The results demonstrate that the model’s line consistently remains above both the all positive and all negative lines within a specific range, indicating higher net benefits and improved model performance. In addition, based on the expression of MRHGs in GSE166502 and the LASSO coefficient, the LASSO risk score for the GSE166502 dataset was calculated, and the ROC curve was drawn using R package pROC. The ROC curve showed that the LASSO risk score for the GSE166502 dataset showed high accuracy between different groups (AUC > 0.9).

### 3.7. Gene Set Enrichment Analysis (GSEA) of the High- and Low-Risk Groups

**Figure 5:**
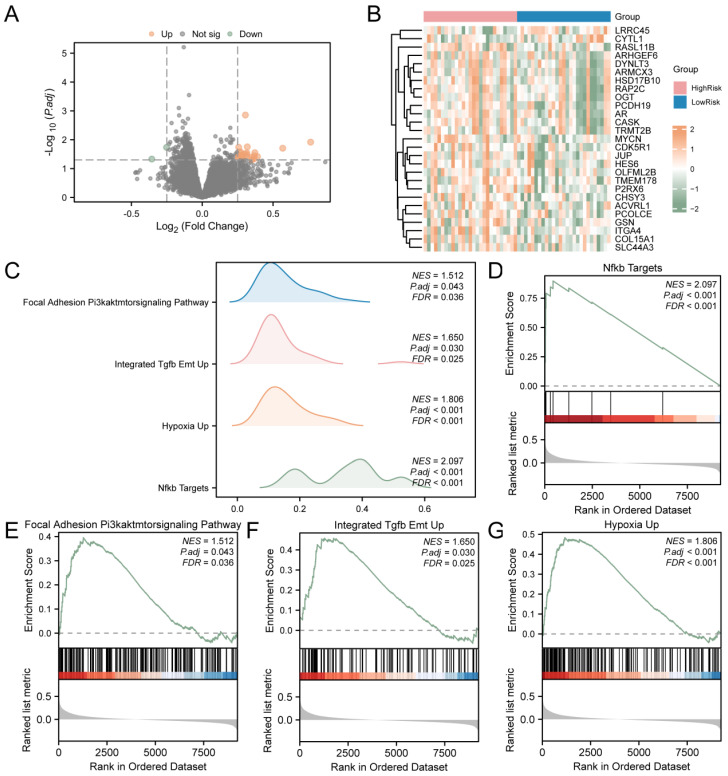
GSEA for Risk Groups. (**A**) DEGs in the high-risk and low-risk groups of T2D samples were analyzed and presented as volcano plots. (**B**) Correlation heat map of DEGs in T2D samples. (**C**) GSEA results of T2D samples presented as four biological function mountain maps. (**D**) GSEA showed that T2D significantly affected NF-κB targets (**D**), focal adhesion and the PI3K–AKT–mTOR signaling pathway (**E**), integrated TGF-β EMT up (**F**), and hypoxia up (**G**). T2D, Type 2 Diabetes; GSEA, Gene Set Enrichment Analysis. The screening criteria of gene set enrichment analysis (GSEA) were adj.*p* < 0.05 and FDR value (q value) < 0.25.

T2D samples were divided into the high-risk and low-risk groups based on LASSO. To assess the gene expression between T2D samples from the high- and low-risk groups, the R package limma was utilized to identify DEGs. The analysis revealed 27 DEGs from T2D samples that met the criteria (|logFC| > 0.25 and *p* value < 0.05). There are 25 down-regulated genes (logFC < −0.25 and *p* value < 0.05). The results are presented as a volcano map (Figure 5A). Then, the intensities and differences in DEGs across various T2D sample groups were analyzed, and a heatmap was drawn using the R package to display the analysis results (Figure 5B).

To assess the impact of gene expression levels on T2D, GSEA was employed to examine the association between gene expression in T2D samples and the BP, cellular components, and the MF involved (Figure 5C). The detailed results are provided in Supplementary Table S5. The findings indicated that all genes in T2D samples were significantly enriched in biologically related functions and signaling pathways, such as NF-κB targets (Figure 5D), focal adhesion and the PI3K–AKT–mTOR signaling pathway (Figure 5E), integrated TGF-β EMT up (Figure 5F), and hypoxia up (Figure 5G).

### 3.8. Gene Set Variation Analysis (GSVA) of the Integrated GEO Dataset

To investigate the difference in the c2.all.v7.5.1.symbols.gmt gene set between T2D samples from the high- and low-risk group, GSVA was conducted on all T2D genes. Detailed information is provided in Supplementary Table S6. Pathways with |logFC| > 0.30 and *p* value < 0.05 were screened, and the findings were presented in a group comparison box plot (Supplementary Figure S5A). The outcomes of GSVA showed that 14 pathways were statistically significant enriched in the high- and the low-risk groups (*p* value < 0.05), including Runx1 and Foxp3 govern the development of regulatory T lymphocytes and Tregs, Runx1 modulates the transcription of genes associated with interleukin signaling, biocarta eryth pathway, weber methylated hcp in fibroblast up, khetchoumian trim24 targets dn, mitochondrial uncoupling, the fatty acid cycling model, motamed response to androgen up, activation of the phototransduction cascade, cytosolic tRNA aminoacylation, Tlr3-mediated Ticam1-dependent programmed cell death, eicosanoid metabolism via the cytochrome p450 monooxygenase pathway, metabolism of amine-derived hormones, and thyroxine biosynthesis. Finally, according to the results of GSVA, the differential expression of 14 pathways with |logFC| > 0.30 and *p* value < 0.05 between the high- and low-risk groups was analyzed and visualized using heat maps (Supplementary Figure S5B).

### 3.9. Correlation and Expression Difference Analysis of MRHGs

First, in order to explore the expression differences of MRHGs in the integrated GEO dataset and GSE166502. The expression levels of 10 MRHGs in the integrated GEO dataset and GSE166502 in T2D and control groups were displayed in a group comparison box diagram (Supplementary Figure S6A,B).

The differential results of GEO are shown in Supplementary Figure S6A. The expression levels of 10 MRHGs in the GEO datasets for T2D were statistically significant (*p* value < 0.001): SLC25A1, KCNK3, PPARG, KIF1C, NPLOC4. TNFAIP8L1, UROD, ACSL3, HSPA1A, and HSP90AB1. The results obtained using GSE166502 (Supplementary Figure S6B) showed that the differences in expression intensities of five MRHGs in the T2D and control groups were statistically significant (*p* value < 0.05). One of these genes was the mitophagy-related hub gene KIF1C. The expression levels of NPLOC4 and HSPA1A in T2D and control groups in the integrated GEO dataset were highly significant from a statistical perspective (*p* value < 0.01); the intensities levels of PPARG and HSP90AB1 in T2D and control groups in GEO were statistically noteworthy (*p* value < 0.05).

Then, based on the complete expression matrix of 10 MRHGs in the integrated GEO dataset and GSE166502, correlation analysis was performed, and the results were visualized using a correlation chord diagram (Supplementary Figure S6C,D). The connection string illustrates the relationship between genes. A wider band and a deeper the color indicate a higher absolute value of the correlation coefficient (r value). The results showed that MRHGs HSP90AB1 and PPARG showed a significant negative correlation in the integrated GEO dataset and GSE166502. HSPA1A and SLC25A1 showed a significant positive correlation in the integrated GEO dataset and GSE166502.

### 3.10. Analysis of Immune Infiltration in T2D

**Figure 6:**
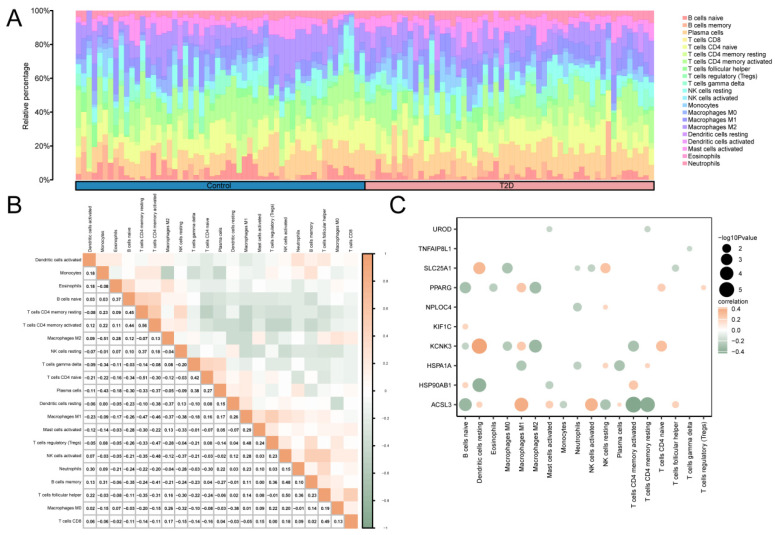
Immune Infiltration Analysis Of Combined Datasets was Performed using the CIBERSORT Algorithm. (**A**) A histogram of the proportion of immune cells in the integrated GEO dataset. (**B**) The correlation heat map of immune cell infiltration abundance in the integrated GEO dataset. (**C**) A point map of the correlation between MRHGs and immune cell infiltration abundance in the integrated GEO dataset. T2D, Type 2 Diabetes. Blue represents the control group, and pink represents the T2D group. Green indicates a negative correlation, and orange indicates a positive correlation. An absolute value of the correlation coefficient (r value) between 0.3 and 0.5 indicates a weak correlation, and a value between 0.5 and 0.8 indicates a moderate correlation.

The correlation between 22 immune cells in the T2D and control groups was calculated using CIBERSORT algorithm using the integrated GEO dataset on T2D. Based on the findings from the immune infiltration analysis, a histogram of the proportion of immune cells in the integrated GEO dataset is shown in Figure 6A. The results of the proportion histogram showed that the enrichment scores of 21 immune cells were greater than zero. Then, the results of analyzing the correlation between the infiltration levels of 21 immune cells, as identified in the immune infiltration analysis, were presented in a correlation point map (Figure 6B). The results showed that activated CD4 memory T cells and resting CD4 memory T cells showed the greatest positive correlation (r value = 0.56). M2 macrophages, monocytes, CD4 naive T cells, and resting CD4 memory T cells showed the greatest negative correlation (r value = −0.51). Finally, the correlation between MRHGs and immune cell infiltration abundance in the T2D dataset was illustrated using the correlation point map (Figure 6C). The results of the correlation plot showed that KCNK3, a mitophagy-related hub gene, had a significant positive correlation with resting dendritic cells. There was a significant negative correlation between ACSL3 and activated CD4 memory T cells.

### 3.11. Construction of Regulatory Network and Prediction of Protein Domain

First, the TF binding to MRHGs was obtained using the ChIPBase database, and the mRNA-TF regulatory network was built and visualized using Cytoscape software (Supplementary Figure S7A). In total, 8 MRHGs and 35 TFs were identified. The results are detailed in Table S2.

Then, miRNAs associated with MRHGs were acquired from the StarBase database, and the mRNA–miRNA regulatory network was developed and visualized using Cytoscape software (Supplementary Figure S7B). Among them, there were 2 MRHGs and 25 miRNAs, and the specific information is shown in Supplementary Table S3.

Next, PPI analysis was performed, and the PPI network of 10 MRHGs was constructed using the STRING database and visualized using Cytoscape software (Supplementary Figure S7C). The results of the PPI network analysis showed that the PPI network contained three related MRHGs: PPARG, HSP90AB1, and HSPA1A. Finally, the SMART database was used to query the protein domains of three related MRHGs (Supplementary Figure S7D–F). Below is the display of the most important secondary domains, and above is the display of the protein domains of MRHGs. Among them, the MRHG PPARG contains domains such as HSP70, HSP90AB1 contains domains such as HATPase_c and HSP90, and HSPA1A contains domains such as PPAR-γ_N, ZnF_C4, and HOLI.

## 4. Discussion

The prevalence and incidence of T2D, accounting for over 90% diabetes cases worldwide, are escalating at an alarming rate. T2D is characterized by a metabolic imbalance in which mitochondria play an indispensable and pivotal role. Mitochondria serve as the primary generators of reactive oxygen species (ROS) and are instrumental in maintaining metabolic processes and regulating numerous cellular functions, including apoptosis [4]. The intricate interplay between mitochondria and inflammation, along with the diverse signaling pathways involved, holds paramount significance. Mitochondria occupy a pivotal position in regulating the immune response by intricately modulating autophagy through various mechanisms, thereby exerting a profound influence on transcription in immune cells [37]. The pivotal role of mitochondria in modulating the inflammatory response associated with T2D underscores its significance. Consequently, a substantial body of evidence underscores the crucial function of mitochondria in IR, making them prime targets for therapeutic interventions aimed at addressing this condition. However, a deeper insight into the regulation of mitochondrial autophagy amd the molecular pathways involved in the development and advancement of T2D is of guiding significance, and this information has the potential to further our understanding of the occurrence and development of T2D. Despite this, there remains a lack of a definite therapeutic target and mechanism for effectively treating this disease. 

This study is the first to explore mitophagy’s involvement in T2D development using bioinformatics. We identified key MRHGs based on the functional enrichment of DEGs, crafting a mitophagy-focused gene signature. Next, we built metabolism–autophagy PPI networks and predicted associated TFs and microRNAs. We also investigated the link between mitophagy and non-coding SNPs. Ultimately, we predicted drugs targeting mitophagy with the potential to curb T2D. Across two expression datasets, we identified 12 relevant genes. CIBERSORT tools have significantly enhanced our ability to dissect the intricate patterns of immune cell infiltration in T2D, providing valuable insights into the disease’s immunological landscape. The strongest positive correlation was observed between activated CD4 memory T cells and resting CD4 memory T cells, suggesting a close relationship between their states. Maximum negative correlations were noted between M2 macrophages and monocytes and between CD4 naive T cells and resting CD4 memory T cells. KCNK3 expression was positively correlated with the sensitivity of resting dendritic cells, whereas there was a significant negative association between ACSL3 and activated CD4 memory T cells. 

In this study, we identified 12 MRDEGs. To explore their biological roles in T2D, we performed enrichment analyses using GO, KEGG, and GSEA methods. GO enrichment analysis revealed that regulation of interferon-gamma-mediated signaling pathway, the modulation of the TGF-β receptor signaling pathway, and ubiquitin-like protein ligase binding were significantly associated with the occurrence and development of T2D. Interferon-gamma (IFN)-γ is a multifunctional cytokine that influences the balance between T helper 1 and T helper 2 cells and modulates cell proliferation and apoptosis. IFN-γ is secreted by T cells, natural killer cells, natural killer T cells, and antigen-presenting cells like macrophages, dendritic cells, and the Be-1 and CD11a^hi^FcγRIII^hi^ subsets of B cells [38]. Additionally, IFN-γ is known to activate several signaling factors, such as NF-κB, MAPK, STAT1, and IRF-1, which regulate its proinflammatory effects. Transforming growth factor-β (TGF-β) was implicated in different phases of pancreatic development. Prior to the initiation of pancreas formation (primary transition), the notochord releases several powerful inducing molecules, including the TGF-β family ligands, which are essential for the onset of pancreas organogenesis [39]. It is suggested that the effect of TGF- β on the growth and development of the pancreas is consistent with the promotion of mitochondrial autophagy by TGF-β in the onset and progression of T2D. Previous research has shown that the NF-κB, MAPK, and STAT pathways could lead to islet β-cell apoptosis, accelerating the development of T2D [40,41,42]. This is the first report on the role of the IFN-γ signal pathway in T2D mitochondrial autophagy, which reflects the reliability of the research results and provides new ideas and targets for T2D therapy.

GSEA was employed to examine the connection between gene expression in T2D samples and the associated biological processes, cellular components, and molecular functions. The results showed that all differentially expressed genes in T2D samples were significantly enriched in the following processes: NF-κB targets, focal adhesion and the PI3K–AKT–mTOR signaling pathway, integrated TGF-β EMT up, hypoxia up, and other biologically related functions and signaling pathways. Signal transduction through Toll-like receptor-4 (TLR4) and tumor necrosis factor-α (TNF-α) receptors activates the NF-κB pathway, leading to decreased mitochondrial respiration and reduced activation of transcription factors that support mitochondrial biosynthesis. Additionally, studies have shown that obesity in rodents and a chronic excess of metabolic fuel to skeletal muscle cells in vitro are linked to enhanced proinflammatory NF-κB signaling and insulin resistance [43]. This is consistent with our findings, indicating that T2D mitophagy is associated with the NF-κB signaling pathway. The phosphatidylinositol 3-kinase (PI3K)/Akt signaling pathway has a significant effect on cell survival, proliferation, and metabolism. Increasing evidence suggests that PI3K/Akt signaling plays a critical role in regulating β-cell mass and function. In T2D, β-cell dysfunction is a very important cause, and mitophagy plays a decisive role in cell functions. Our results show that all differentially expressed genes in T2D samples are enriched in PI3K and other pathways, which is consistent with previous studies.

We identified 12 hub genes by constructing the PPI network and mainly focused five genes, including SLC25A1, PPARG, NPLOC4, ACSL3, and HSPA1A. SLC25A1 is the mitochondrial citrate carrier that exports citrate out of the mitochondrial matrix [39]. The absence of SLC25A1 reduces the function of the respiratory chain in vitro, indicating that SLC25A1 may also have a direct role in regulating mitochondrial function [44,45]. Previously, it has been suggested that SLC25A1 acts as a metabolic oncogene, although its significance in cancer treatment remains unclear [36,37]. Previous studies have shown that SLC25A1 directly regulates mitochondrial function and plays a crucial role in maintaining the mitochondrial pool and the redox equilibrium of citric acid in non-small cell lung cancer. This study found for the first time that SLC25A1 plays a key role in mitochondrial autophagy in T2D, indicating that SLC25A1 is a protein that may play an important role in the occurrence, development, and treatment of T2D.

PPAR-γ belongs to the family of PPARs and is a pivotal nuclear transcription factor that modulates gene expression in response to peroxisome proliferators and fatty acid metabolites [39]. PPAR signaling is essential not only for lipid metabolism and glucose homeostasis but also for influencing immune response, cell growth, development, differentiation, apoptosis, and cell movement [46]. It has been shown that PPAR-γ influences lipid balance by controlling the transcription of genes related to lipid metabolism and storage. Given that dyslipidemia is a significant risk factor for atherosclerosis, managing blood lipid levels therapeutically could help protect against cardiovascular disease in individuals with T2D [47]. Activating PPAR-γ in adipocytes enhances the regulated release of adipocytokines like adiponectin and leptin, which support insulin action in peripheral tissues and help preserve insulin sensitivity [40]. This is consistent with our results, indicating that PPAR-γ is involved in mitochondrial autophagy, regulates glucose metabolism, and affects insulin secretion in the process leading to T2D.

ACSL3 is a member of the long chain acyl-CoA synthetase family and functions as a cholesterol biosynthesis enzyme. ACSL3 plays a role in facilitating LPCAT4’s effects on cholesterol metabolism [39]. It reported that ACSL3 promotes resistance to ferroptosis by substituting PUFAs in PLs with monounsaturated fatty acids (MUFAs) [48]. Previous studies have pointed out that ACSL3 is related to lipid metabolism. Our study found for the first time that ACSL3 is related to glucose metabolism and T2D mitochondrial autophagy, which provides a new idea for T2D therapy.

LASSO logistic and forest plot regressions were used to verify the selected mitophagy-related differentially expressed hub genes involved in the T2D. These genes include SLC25A1, KCNK3, PPARG, KIF1C, NPLOC4, TNFAIP8L1, UROD, ACSL3, HSPA1A, and HSP90AB1. These MRDEGs are meaningful. Hub genes identified from the combined GEO datasets showed that the expression of mitophagy-related hub genes SLC25A1 and PPARG was significantly more effective than other factors in the diagnostic model of T2D. The effect of ACSL3 expression on the diagnostic model of T2D was significantly lower than that of other variables. The results of mitophagy-related hub genes obtained using the dataset GSE166502 showed that the mitophagy-related hub gene NPLOC4 was significantly more effective than other factors in the diagnostic model for T2D. The value of HSPA1A expression in the diagnosis model of T2D was significantly lower than that of other variables.

There are some limitations in the current study. The MRHG analysis results were derived from data obtained from T2D and normal muscle tissues based on a small sample size; thus, our findings require validation with a larger T2D cohort. Unfortunately, due to technical limitations, we were unable to obtain muscle tissue from T2D patients in our hospital, and verification could only be performed experimentally using blood samples. Another limitation is that this study should include fundamental experiments to validate the expression of the identified hub genes and diagnostic marker genes. For instance, the functions of these hub genes and markers should be thoroughly investigated using techniques such as real-time PCR, immunohistochemistry, and immunofluorescence. Therefore, a more comprehensive investigation is needed moving forward.

## 5. Conclusions

In this study, 12 MRHGs linked to T2D were identified using bioinformatic and machine learning methods. These genes may influence the development and prognosis of T2D by modulating mitophagy. The results enhance our understanding of T2D and could inform future treatment strategies for this condition.

## Figures and Tables

**Table 1:** GEO Microarray Chip Information.

	GSE166467	GSE166652	GSE106090
Platform	GPL10558	GPL13534	GPL10558
Species	Homo sapiens	Homo sapiens	Homo sapiens
Tissue	Muscle Tissue	Muscle Tissue	Muscle Tissue
Samples in T2D Group	26	28	26
Samples in Control Group	26	28	26
Reference	PMID: 33893273	PMID: 33893273	PMID: 31853997

T2D, Type 2 Diabetes; GEO, Gene Expression Omnibus.

## Data Availability

All data generated or analyzed during this study are included in this published article and Supplementary Materials.

## Supplementary Materials

The following supporting information can be downloaded at: https://www.mdpi.com/article/10.3390/cimb46090619/s1, Figure S1: Flow Chart for the Comprehensive Analysis of MRDEGs; Figure S2: Batch Effects Removal of GSE166467 and GSE166652; (A,B). Distribution boxplot of the integrated GEO dataset (Combined Datasets) before de-batching (A) and after de-batching (B). (C,D). PCA diagram of the integrated GEO dataset (Combined Datasets) before de-batching (C) and after de-batching (D). PCA, Principal Component Analysis; t2D, Type 2 Diabetes. Orange is type 2 diabetes (T2D) data set GSE166467, and green is type 2 diabetes (T2D) data set GSE166652. Figure S3: GSVA for Combined Datasets; A-B. Results of gene set variation analysis (GSVA) Grouped comparison box plots (A) and complex numerical heat maps (B) in the type 2 diabetes (T2D) group and the control (Control) group. T2D, Type 2 Diabetes; gSVA, Gene Set Variation Analysis. Blue was the control group and pink was the type 2 diabetes mellitus (T2D) group. *** represents *p* value 0.00 and adj.*p* < 0.05. Figure S4: Diagnostic and Validation Analysis of T2D; (A). Nomogram of mitophagy-related hub genes in the integrated GEO data sets in the diagnostic model of type 2 diabetes mellitus (T2D). (B). Nomogram of mitophagy-related hub genes in the data set GSE166502 of type 2 diabetes (T2D) diagnostic model. The (C,D). 2 diabetes (T2D) diagnosis model is based on the Calibration Curve (C) and Decision Curve Analysis (DCA) of mitophagy-related hub genes in the integrated GEO dataset. The ROC curve of (E). LASSO Risk Score in the integrated GEO dataset. The (F,G). 2 diabetes (T2D) diagnosis model is based on the Calibration Curve (F) and Decision Curve Analysis (DCA) (G) of the mitophagy-related hub genes in the data set GSE166502. The ROC curve of LASSO risk score (Risk Score) in the data set GSE166502 (H). The ordinate is the net income, and the abscissa is the probability threshold or threshold probability. PE, Pre-Eclamptic; dCA, Decision Curve Analysis. When AUC is above 0.9, it has higher accuracy. Figure S5: GSVA for Risk Group; (A,B). Gene Set Variability Analysis (GSVA) results Grouped comparison box plots (A) and complex numerical heat maps (B) in the high-risk (High Risk) group and the low-risk (Low Risk) group. T2D, Type 2 Diabetes; gSVA, Gene Set Variation Analysis. Blue was low risk group and pink was high risk group. *** represents p value 0.30 and *p* value < 0.05. Figure S6: Correlation and Expression Difference Analysis for Hub Genes; (A). Grouping comparison box plots of mitophagy-related hub genes in the integrated GEO dataset (Combined Datasets). (B). Group comparison box plot of mitophagy-related hub genes in dataset GSE166502. (C). The correlation circle diagram of mitophagy-related hub genes in the integrated GEO dataset. (D). The correlation circle diagram of mitophagy-related hub genes in the data set GSE166502. Blue was the control group and pink was the type 2 diabetes mellitus (T2D) group. * represents *p* value < 0.05, which is statistically significant; ** represents *p* value < 0.01, with a high degree of statistical significance; *** represents *p* value < 0.001, which is statistically significant. (A). Correlation coefficient (r value) Orange is positively correlated, green is negatively correlated; the connection string represents the correlation between genes. The wider the band, the deeper the color, and the greater the absolute value of the correlation coefficient (r value). Figure S7: Regulatory Network and Protein Domains of Hub Genes; (A). mRNA-TF regulatory network of mitophagy-related hub genes. (B). mRNA-miRNA regulatory network of mitophagy-related hub genes. (C). Protein-protein interaction network (PPI Network) of mitophagy-related hub genes. (D–F). Predictive protein domains of mitophagy-related hub genes PPARG (D), HSP90AB1 (E) and HSPA1A (F). TF, Transcription Factor. Orange is mRNA, green is TF, and blue is miRNA. Table S1: GEO Microarray Chip Information; Table S2: Results of GO and KEGG Enrichment Analysis for MRDEGs; Table S3: Results of GSEA for Combined Datasets; Table S4: Results of GSVA for Combined Datasets; Table S5: Results of GSEA for Risk Group; Table S6: Results of GSVA for Risk Group.
