# Supplementary material for: Integrative Analyses of Mitophagy-Related Genes and Mechanisms Associated with Type 2 Diabetes in Muscle Tissue
Source: Curr Issues Mol Biol. 2024 Sep 18;46(9):10411–29. doi: 10.3390/cimb46090619 (PMC11430763; doi:10.3390/cimb46090619)
Supplement: Supplementary file 1 [file cimb-46-00619-s001.zip › cimb-3148247-supplementary.pdf]

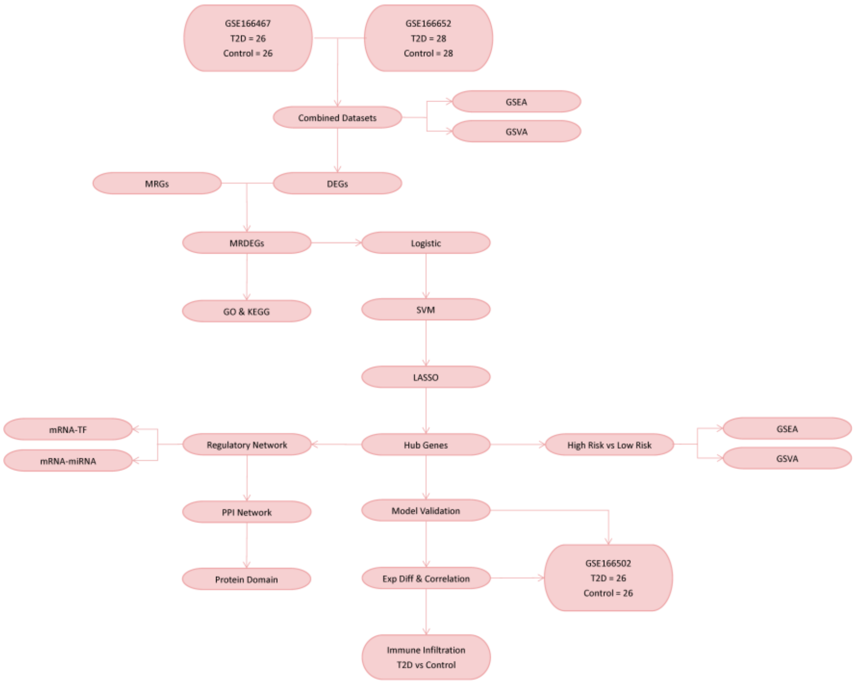

Supplementary Figure S1. Flow Chart for the Comprehensive Analysis of MRDEGs.

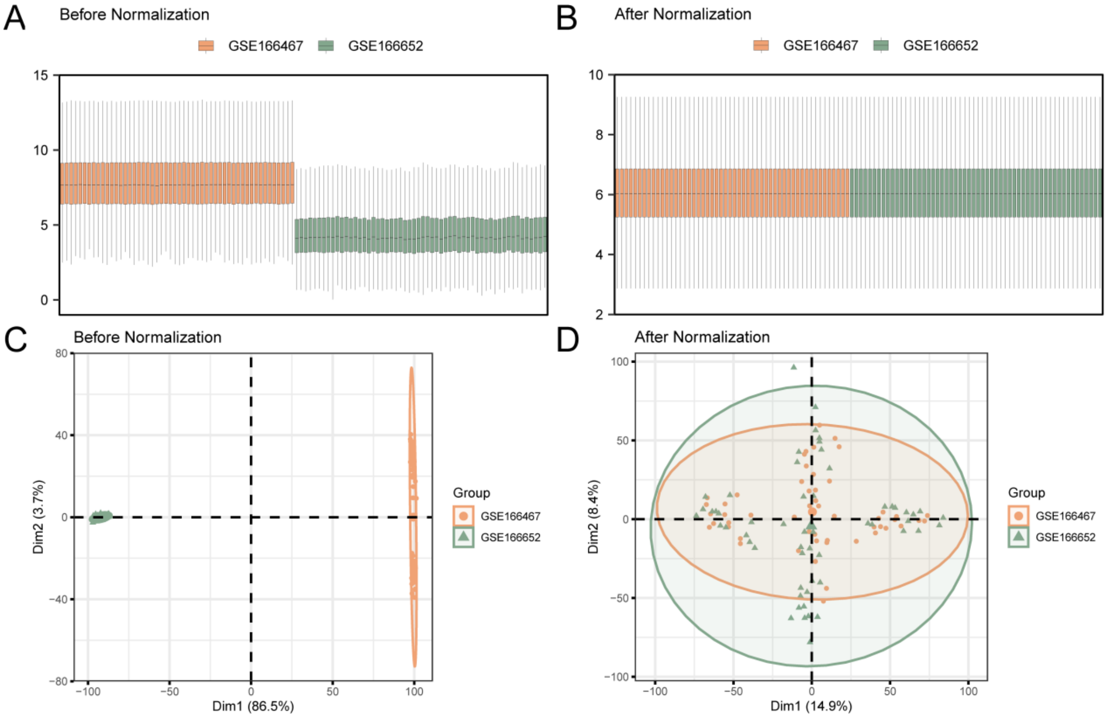

Supplementary Figure S2. Batch Effects Removal of GSE166467 and GSE166652; A-B. Distribution boxplot of the integrated GEO dataset (Combined Datasets) before de-batching (A) and after de-batching (B). C-D.PCA di-agram of the integrated GEO dataset (Combined Datasets) before de-batching (C) and after de-batching (D). PCA, Principal Component Analysis; t2D, Type 2 Diabetes. Orange is type 2 di-abetes (T2D) data set GSE166467, and green is type 2 diabetes (T2D) data set GSE166652.

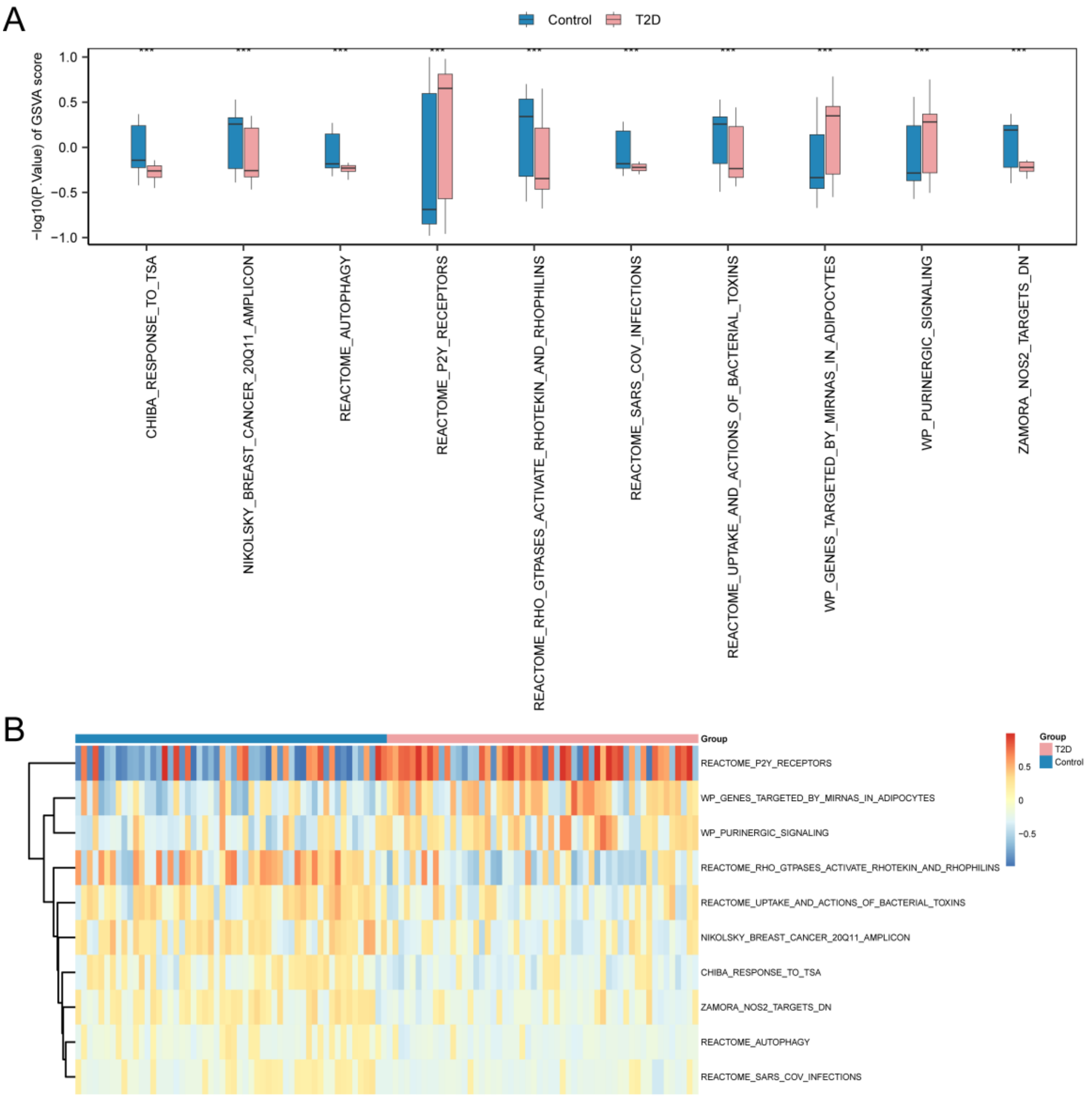

Supplementary Figure S3. GSVA for Combined Datasets; A-B. Results of gene set variation analysis (GSVA) Grouped com-parison box plots (A) and complex numerical heat maps (B) in the type 2 diabetes (T2D) group and the control (Control) group. T2D, Type 2 Diabetes; gSVA, Gene Set Variation Analysis. Blue was the control group and pink was the type 2 diabetes mellitus (T2D) group. * * * represents p value 0.00 and adj.p < 0.05.

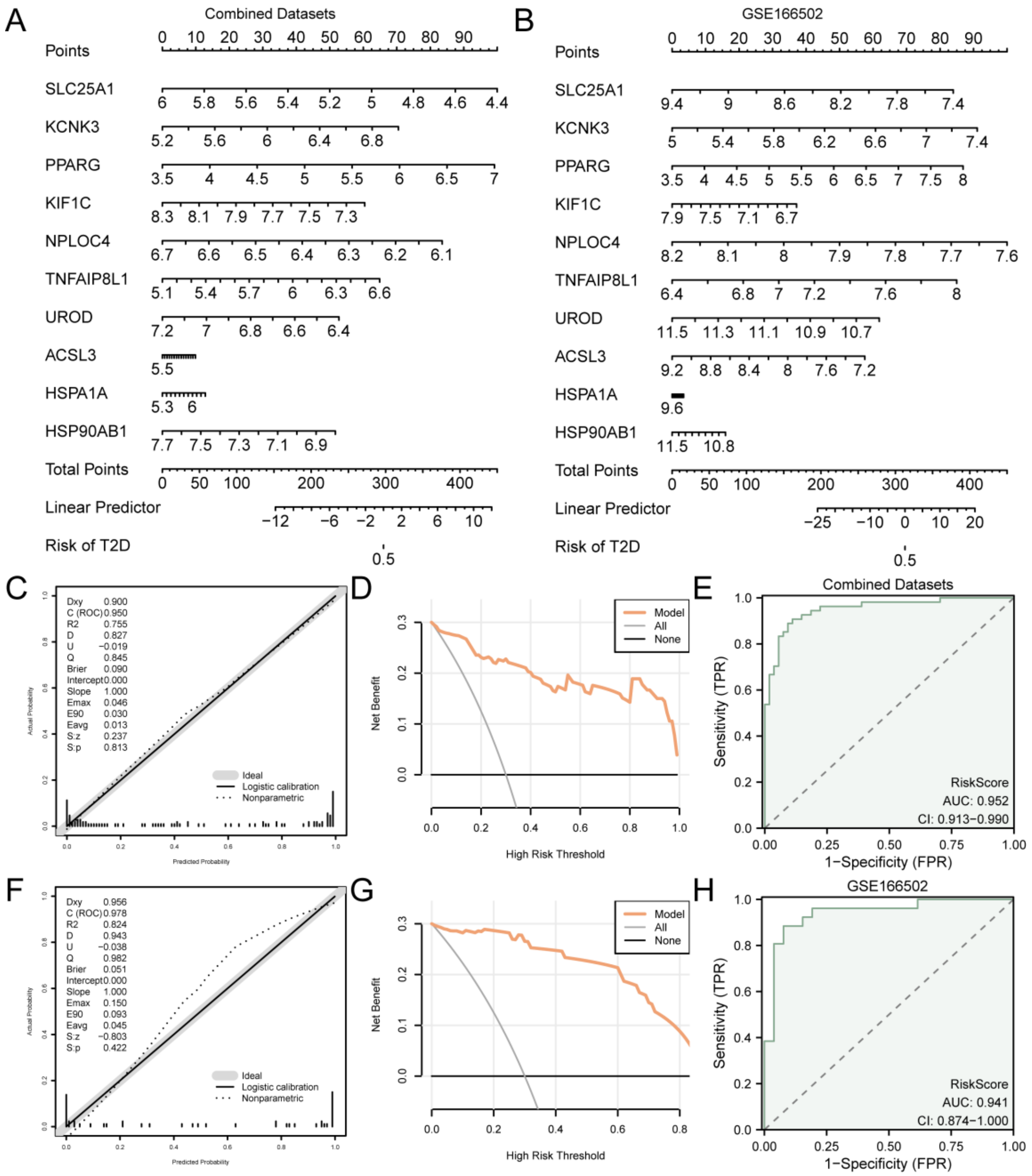

Supplementary Figure S4. Diagnostic and Validation Analysis of T2D; A. Nomogram of mitophagy-related hub genes in the integrated GEO data sets in the diagnostic model of type 2 diabetes mellitus (T2D). B. Nomogram of mitophagy-related hub genes in the data set GSE166502 of type 2 diabetes (T2D) diagnostic model. The C-D.2 diabetes (T2D) diagnosis model is based on the Calibration Curve (C) and Decision Curve Analysis (DCA) of mitophagy-related hub genes in the integrated GEO dataset. The ROC curve of E. LASSO Risk Score in the integrated GEO dataset. The F-G.2 diabetes (T2D) diagnosis model is based on the Calibration Curve (F) and Decision Curve Analysis (DCA) (G) of the mitophagy-related hub genes in the data set GSE166502. The ROC curve of LASSO risk score (Risk Score) in the data set GSE166502(H). The ordinate is the net income, and the abscissa is the probability threshold or threshold probability. PE, Pre-Eclamptic; dCA, Decision Curve Analysis. When AUC is above 0.9, it has higher accuracy.

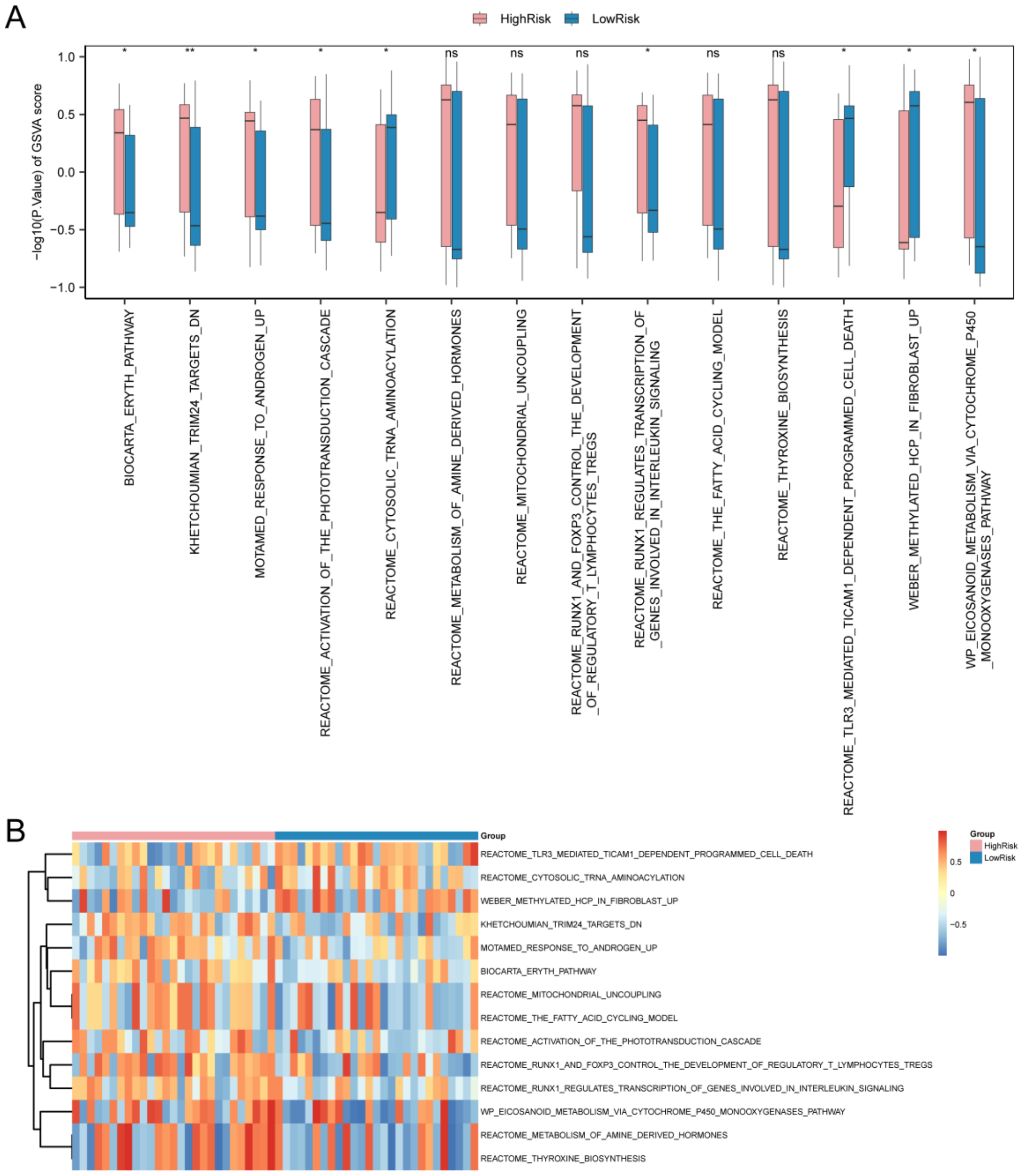

Supplementary Figure S5. : GSVA for Risk Group; A-B. Gene Set Variability Analysis (GSVA) results Grouped comparison box plots (A) and complex numerical heat maps (B) in the high-risk (High Risk) group and the low-risk (Low Risk) group. T2D, Type 2 Diabetes; gSVA, Gene Set Variation Analysis. Blue was low risk group and pink was high risk group. * * * represents p value 0.30 and p value < 0.05.

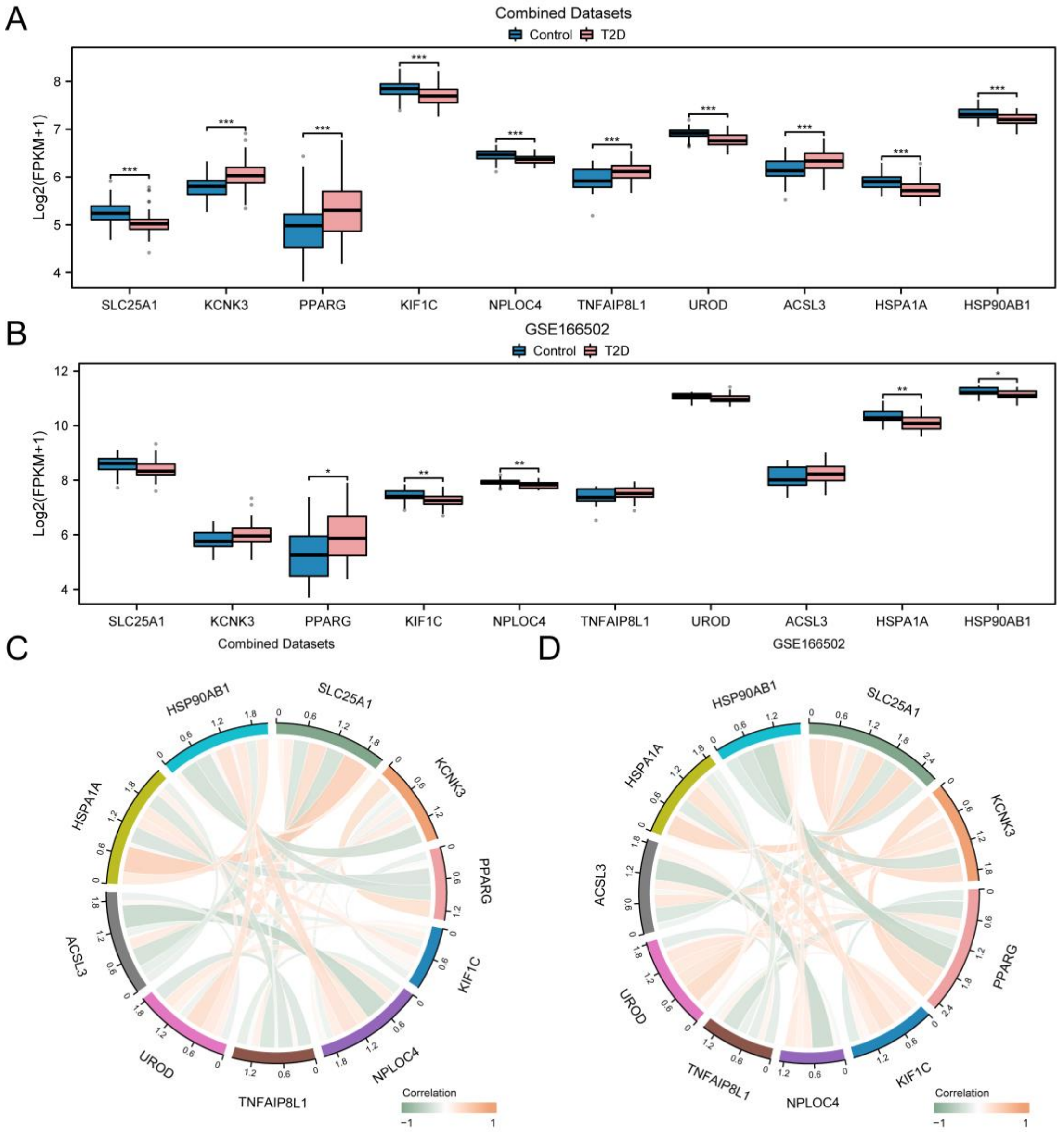

Supplementary Figure S6. Correlation and Expression Difference Analysis for Hub Genes; A. Grouping comparison box plots of mitophagy-related hub genes in the integrated GEO dataset (Combined Datasets). B. Group comparison box plot of mitophagy-related hub genes in dataset GSE166502. C. The correlation circle diagram of mitophagy-related hub genes in the integrated GEO dataset. D. The correlation circle diagram of mitophagy-related hub genes in the data set GSE166502. Blue was the control group and pink was the type 2 diabetes mellitus (T2D) group. * represents p value < 0.05, which is statistically significant; * * represents p value < 0.01, with a high degree of statistical significance; * * * represents p value < 0.001, which is statistically significant. A. Correlation coefficient (r value) Orange is pos-itively correlated, green is negatively correlated; the connection string represents the correlation between genes. The wider the band, the deeper the color, and the greater the absolute value of the correlation coefficient (r value).

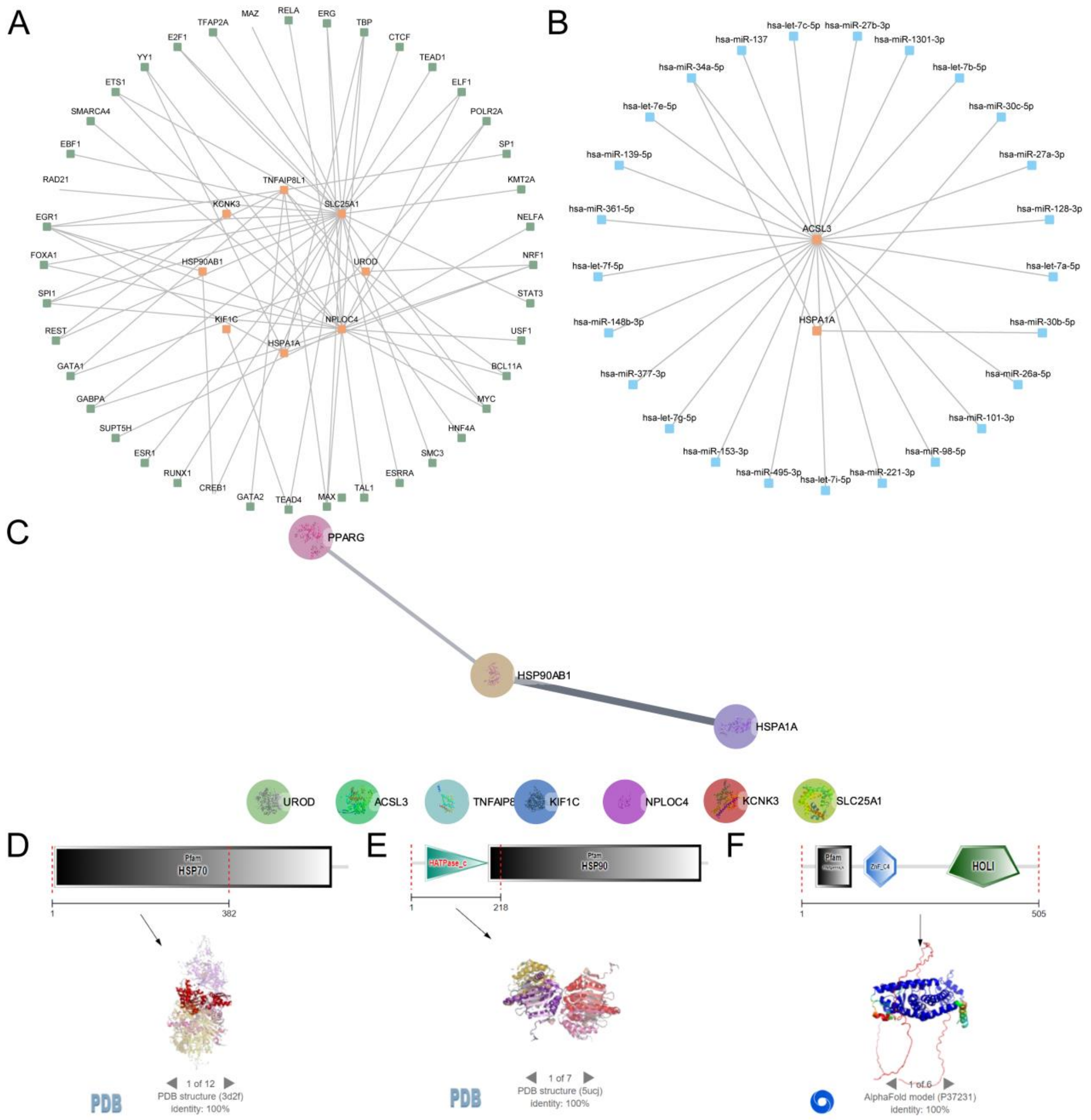

Supplementary Figure S7. Regulatory Network and Protein Domains of Hub Genes; A. mRNA-TF regulatory network of mitophagy-related hub genes. B. mRNA-miRNA regulatory network of mitophagy-related hub genes. C. Protein-protein interaction network (PPI Network) of mitophagy-related hub genes. D-F. Predictive protein domains of mitophagy-related hub genes PPARG (D), HSP90AB1 (E) and HSPA1A (F). TF, Transcription Factor. Orange is mRNA, green is TF, and blue is miRNA. Table S1: GEO Microarray Chip Information; Table S2: Results of GO and KEGG Enrichment Analysis for MRDEGs; Table S3: Results of GSEA for Combined Datasets ;Table S4: Results of GSVA for Com-bined Datasets; Table S5: Results of GSEA for Risk Group; Table S6: Results of GSVA for Risk Group.

Supplementary Table S1. GEO Microarray Chip Information

	GSE166467	GSE166652	GSE106090
Platform	GPL10558	GPL13534	GPL10558
Species	Homo sapiens	Homo sapiens	Homo sapiens
Tissue	Muscle Tissue	Muscle Tissue	Muscle Tissue
Samples in T2D Group	26	28	26
Samples in Control Group	26	28	26
Reference	PMID:	PMID:	PMID:

T2D，Type 2 Diabetes；GEO，Gene Expression Omnibus。

Supplementary Table S2. Results of GO and KEGG Enrichment Analysis for MRDEGs

ONTOLOGY	ID	Description	GeneRatio	BgRatio	pvalue	p.adjust	qvalue
BP	GO:0097201	negative regulation of transcription from RNA polymerase II promoter in response to stress	2/12	12/18800	2.46E-05	6.55E-03	3.25E-03
BP	GO:0060330	regulation of response to interferon-gamma	2/12	16/18800	4.46E-05	6.55E-03	3.25E-03
BP	GO:0060334	regulation of interferon-gamma-mediated signaling pathway	2/12	16/18800	4.46E-05	6.55E-03	3.25E-03
BP	GO:0017015	regulation of transforming growth factor beta receptor signaling pathway	3/12	130/18800	6.79E-05	6.55E-03	3.25E-03
BP	GO:1902236	negative regulation of endoplasmic reticulum stress-induced intrinsic apoptotic signaling pathway	2/12	20/18800	7.05E-05	6.55E-03	3.25E-03
MF	GO:0031625	ubiquitin protein ligase binding	4/12	298/18410	3.01E-05	2.03E-03	1.01E-03
MF	GO:0044389	ubiquitin-like protein ligase binding	4/12	317/18410	3.83E-05	2.03E-03	1.01E-03
MF	GO:0008022	protein C-terminus binding	3/12	179/18410	1.86E-04	6.07E-03	3.01E-03
MF	GO:0097718	disordered domain specific binding	2/12	35/18410	2.29E-04	6.07E-03	3.01E-03
MF	GO:0044183	protein folding chaperone	2/12	43/18410	3.47E-04	7.35E-03	3.65E-03
KEGG	hsa04141	Protein processing in endoplasmic reticulum	3/8	171/8164	4.68E-04	1.97E-02	1.54E-02
KEGG	hsa05417	Lipid and atherosclerosis	3/8	215/8164	9.15E-04	1.97E-02	1.54E-02
KEGG	hsa03320	PPAR signaling pathway	2/8	75/8164	2.25E-03	2.61E-02	2.05E-02
KEGG	hsa04612	Antigen processing and presentation	2/8	78/8164	2.43E-03	2.61E-02	2.05E-02

GO，Gene Ontology；BP，Biological Process；MF，Molecular Function；KEGG，Kyoto Encyclopedia of Genes and Genomes；MRDEGs，Mitophagy-Related Differentially Expressed Genes。

Supplementary Table S3. Results of GSEA for Combined Datasets

ID	setSize	enrichmentScore	NES	pvalue	p.adjust	qvalues
NABA_COLLAGENS	23	7.81E-01	2.24E+00	2.01E-06	5.10E-04	4.84E-04
REACTOME_COLLAGEN_CHAIN_TRIMERIZATION	23	7.81E-01	2.24E+00	2.01E-06	5.10E-04	4.84E-04
REACTOME_MUSCLE_CONTRACTION	102	5.92E-01	2.24E+00	3.94E-09	5.08E-06	4.81E-06
PID_INTEGRIN1_PATHWAY	51	6.68E-01	2.23E+00	1.11E-06	4.34E-04	4.11E-04
NABA_CORE_MATRISOME	130	5.47E-01	2.16E+00	1.99E-08	1.35E-05	1.28E-05
REACTOME_STRIATED_MUSCLE_CONTRACTION	29	7.12E-01	2.13E+00	1.85E-05	3.73E-03	3.54E-03
PID_SYNDECAN_1_PATHWAY	30	7.08E-01	2.12E+00	7.98E-06	1.80E-03	1.71E-03
WP_STRIATED_MUSCLE_CONTRACTION_PATHWAY	32	6.93E-01	2.08E+00	2.02E-05	3.73E-03	3.54E-03
REACTOME_COLLAGEN_FORMATION	47	6.16E-01	2.02E+00	4.68E-05	7.88E-03	7.47E-03
NABA_PROTEOGLYCANS	13	8.22E-01	2.02E+00	5.05E-05	7.88E-03	7.47E-03
REACTOME_INTEGRIN_CELL_SURFACE_INTERACTIONS	50	6.04E-01	2.01E+00	6.87E-05	9.29E-03	8.81E-03
REACTOME_COLLAGEN_DEGRADATION	33	6.63E-01	2.01E+00	1.03E-04	1.23E-02	1.17E-02
REACTOME_COLLAGEN_BIOSYNTHESIS_AND_MODIFYING_ENZYMES	36	6.48E-01	2.00E+00	6.81E-05	9.29E-03	8.81E-03
NABA_BASEMENT_MEMBRANES	25	6.83E-01	1.98E+00	3.40E-04	3.17E-02	3.00E-02
REACTOME_EXTRACELLULAR_MATRIX_ORGANIZATION	162	4.82E-01	1.94E+00	7.56E-07	3.84E-04	3.64E-04
REACTOME_DEGRADATION_OF_THE_EXTRACELLULAR_MATRIX	65	5.40E-01	1.91E+00	2.94E-04	2.98E-02	2.83E-02
KEGG_ARRHYTHMOGENIC_RIGHT_VENTRICULAR_CARDIOMYOPATHY_ARVC	44	5.87E-01	1.90E+00	3.59E-04	3.17E-02	3.00E-02
WP_ARRHYTHMOGENIC_RIGHT_VENTRICULAR_CARDIOMYOPATHY	44	5.87E-01	1.90E+00	3.59E-04	3.17E-02	3.00E-02
REACTOME_SYNTHESIS_OF_PROSTAGLANDINS_PG_AND_THROMBOXANES_TX	10	8.21E-01	1.88E+00	4.99E-04	4.05E-02	3.84E-02

KEGG_HYPERTROPHIC_CARDIOMYOPATHY_HCM	53	5.52E-01	1.87E+00	4.05E-04	3.42E-02	3.24E-02

GSEA，Gene Set Enrichment Analysis。

Supplementary Table S4. Results of GSVA for Combined Datasets

ID	logFC	AveExpr	p value	adj.p value
WP_GENES_TARGETED_BY_MIRNAS_IN_ADIPOCYTES	3.77E-01	-2.19E-02	8.58E-07	5.42E-03
REACTOME_RHO_GTPASES_ACTIVATE_RHOTEKIN_AND_RHOPHILINS	-3.73E-01	-1.36E-02	5.99E-06	1.89E-02
ZAMORA_NOS2_TARGETS_DN	-1.96E-01	-4.60E-02	1.61E-05	2.19E-02
CHIBA_RESPONSE_TO_TSA	-2.00E-01	-1.07E-01	1.99E-05	2.19E-02
REACTOME_SARS_COV_INFECTIONS	-1.49E-01	-1.24E-01	2.03E-05	2.19E-02
REACTOME_P2Y_RECEPTORS	5.96E-01	6.66E-04	2.07E-05	2.19E-02
NIKOLSKY_BREAST_CANCER_20Q11_AMPLICON	-2.36E-01	-3.87E-03	4.01E-05	3.41E-02
REACTOME_UPTAKE_AND_ACTIONS_OF_BACTERIAL_TOXINS	-2.40E-01	8.60E-03	4.31E-05	3.41E-02
REACTOME_AUTOPHAGY	-1.27E-01	-1.54E-01	6.30E-05	4.43E-02
WP_PURINERGIC_SIGNALING	2.75E-01	-1.15E-02	7.20E-05	4.55E-02

GSVA，Gene Set Variation Analysis。

Supplementary Table S5. Results of GSEA for Risk Group

ID	setSize	enrichmentScore	NES	pvalue	p.adjust	qvalues
DELASERNA_MYOD_TARGETS_UP	65	7.89E-01	2.64E+00	1.00E-10	2.06E-08	1.70E-08
KUNINGER_IGF1_VS_PDGFB_TARGETS_UP	54	7.97E-01	2.59E+00	1.00E-10	2.06E-08	1.70E-08
REN_ALVEOLAR_RHABDOMYOSARCOMA_UP	69	7.60E-01	2.56E+00	1.00E-10	2.06E-08	1.70E-08
REACTOME_MUSCLE_CONTRACTION	102	7.12E-01	2.55E+00	1.00E-10	2.06E-08	1.70E-08
REACTOME_STRIATED_MUSCLE_CONTRACTION	29	8.78E-01	2.54E+00	1.00E-10	2.06E-08	1.70E-08
WP_STRIATED_MUSCLE_CONTRACTION_PATHWAY	32	8.60E-01	2.54E+00	1.00E-10	2.06E-08	1.70E-08
EBAUER_MYOGENIC_TARGETS_OF_PAX3_FOXO1_FUSION	40	8.21E-01	2.53E+00	1.00E-10	2.06E-08	1.70E-08
RICKMAN_HEAD_AND_NECK_CANCER_F	36	8.36E-01	2.52E+00	1.00E-10	2.06E-08	1.70E-08
EBAUER_TARGETS_OF_PAX3_FOXO1_FUSION_UP	125	6.76E-01	2.49E+00	1.00E-10	2.06E-08	1.70E-08
DAVICIONI_TARGETS_OF_PAX_FOXO1_FUSIONS_DN	45	7.71E-01	2.41E+00	7.70E-10	1.14E-07	9.39E-08
PID_INTEGRIN1_PATHWAY	51	7.37E-01	2.37E+00	1.08E-09	1.55E-07	1.28E-07
BOQUEST_STEM_CELL_UP	190	6.01E-01	2.31E+00	1.00E-10	2.06E-08	1.70E-08
KEGG_HYPERTROPHIC_CARDIOMYOPATHY_HCM	53	7.15E-01	2.31E+00	1.18E-08	1.33E-06	1.10E-06
ANASTASSIOU_MULTICANCER_INVASIVENESS_SIGNATURE	56	7.08E-01	2.30E+00	9.61E-09	1.11E-06	9.16E-07
KEGG_DILATED_CARDIOMYOPATHY	55	7.03E-01	2.29E+00	1.29E-08	1.42E-06	1.17E-06
VECCHI_GASTRIC_CANCER_ADVANCED_VS_EARLY_UP	114	6.26E-01	2.27E+00	1.00E-10	2.06E-08	1.70E-08
WANG_NFKB_TARGETS	11	8.97E-01	2.10E+00	5.93E-06	2.53E-04	2.09E-04
MANALO_HYPOXIA_UP	151	4.83E-01	1.81E+00	2.15E-05	7.30E-04	6.02E-04
FOROUTAN_INTEGRATED_TGFB_EMT_UP	104	4.58E-01	1.65E+00	2.34E-03	3.02E-02	2.49E-02
WP_FOCAL_ADHESION_PI3KAKTMTORSIGNALING_PATHWAY	184	3.95E-01	1.51E+00	3.66E-03	4.33E-02	3.57E-02

Supplementary Table S6. Results of GSVA for Risk Group

ID	logFC	AveExpr	p value	adj.p value
REACTOME_RUNX1_AND_FOXP3_CONTROL_THE_DEVELOPMENT_OF_REGULATORY_T_LYMPHOCYTES_TREGS	-4.46E-01	4.11E-02	1.05E-02	9.67E-01
REACTOME_RUNX1_REGULATES_TRANSCRIPTION_OF_GENES_INVOLVED_IN_INTERLEUKIN_SIGNALING	-3.42E-01	3.31E-02	1.32E-02	9.67E-01
BIOCARTA_ERYTH_PATHWAY	-3.16E-01	-2.27E-02	1.34E-02	9.67E-01
WEBER_METHYLATED_HCP_IN_FIBROBLAST_UP	4.27E-01	-2.93E-02	1.40E-02	9.67E-01
KHETCHOUMIAN_TRIM24_TARGETS_DN	-3.63E-01	-7.87E-03	1.43E-02	9.67E-01
REACTOME_MITOCHONDRIAL_UNCOUPLING	-3.79E-01	-2.65E-02	2.19E-02	9.67E-01
REACTOME_THE_FATTY_ACID_CYCLING_MODEL	-3.79E-01	-2.65E-02	2.19E-02	9.67E-01
MOTAMED_RESPONSE_TO_ANDROGEN_UP	-3.03E-01	3.31E-03	2.76E-02	9.67E-01
REACTOME_ACTIVATION_OF_THE_PHOTOTRANSDUCTION_CASCADE	-3.21E-01	-4.60E-02	3.07E-02	9.67E-01
REACTOME_CYTOSOLIC_TRNA_AMINOACYLATION	3.02E-01	-1.41E-02	3.52E-02	9.67E-01
REACTOME_TLR3_MEDIATED_TICAM1_DEPENDENT_PROGRAMMED_CELL_DEATH	3.23E-01	5.97E-02	4.00E-02	9.67E-01
WP_EICOSANOID_METABOLISM_VIA_CYTOCHROME_P450_MONOOXYGENASES_PATHWAY	-4.01E-01	-2.31E-02	4.57E-02	9.67E-01
REACTOME_METABOLISM_OF_AMINE_DERIVED_HORMONES	-3.93E-01	-4.20E-02	4.99E-02	9.67E-01
REACTOME_THYROXINE_BIOSYNTHESIS	-3.93E-01	-4.20E-02	4.99E-02	9.67E-01

GSVA，Gene Set Variation Analysis。
